# Experiences of foreign medical graduates (FMGs), international medical graduates (IMGs) and overseas trained graduates (OTGs) on entering developing or middle-income countries like South Africa: a scoping review

**DOI:** 10.1186/s12960-019-0343-y

**Published:** 2019-01-21

**Authors:** M. I. Motala, J. M. Van Wyk

**Affiliations:** Clinical and Professional Practice, Nelson R. Mandela School of Medicine, College of Health Sciences, University of KwaZulu-Natal, Room 12, Nelson R. Mandela School of Medicine, Umbilo Road, Durban, 4000 South Africa

**Keywords:** Foreign medical graduates, Barriers, Facilitators, Acculturation, Adjustment, Experiences, High- and middle-income countries

## Abstract

**Background:**

Foreign medical graduates (FMGs) have continued to render effective health care services to underserved communities in many high- and middle-income countries. In rural and disadvantaged areas of South Africa, FMGs have alleviated the critical shortage of doctors. FMGs experience challenges to adjust to new working environments as they have studied and obtained their medical qualifications in a country that differs from the one where they eventually choose to practise.

**Objectives:**

This scoping review synthesises literature about the experiences of FMGs upon entering a host country and the factors that facilitate their adjustment to the new context.

**Methods:**

The systematic review was performed to analyse articles from an initial scoping of published literature on the experiences and adjustment of FMGs between 2000 and 2016. Searches were conducted through MEDLINE and PUBMED on keywords that included “foreign medical graduates”, “experiences” “adjustment”, “adaptation” and “assimilation”. The database searches yielded 268 articles and a further 3 were identified through other sources. The number of articles was reduced to 20 after the removal of duplicates and the application of the exclusion criteria. A qualitative thematic analysis was performed.

**Results:**

The searches revealed an overall lack of studies on the experiences and adjustment of FMGs from the African continent. FMGs faced professional barriers, lacked country-specific knowledge and experienced stress when practising in a new location. They attributed their successful adjustment to innate personal characteristics including a persistent attitude and the use of various coping strategies. Other facilitating factors included early orientation and professional and personal support.

**Conclusion:**

The review highlighted the need for research from developing and middle-income countries and for an increased awareness of the challenges and enablers to help FMGs adjust to new clinical settings.

## Introduction

The effective functioning of health care systems of many high-income countries (HICs) depends on the regular intake of FMGs. A study conducted in 2005 reported that FMGs comprised between 23 and 28% of the medical workforce in Australia, Canada, the United Kingdom and the United States of America [1]. In these countries, FMGs are sourced to work among the most vulnerable communities who often live in rural or peripheral geographic locations. According to a study conducted in 2007, up to 65% of FMGs work in locations outside capital cities [2]. Compensation to FMGs usually includes financial incentives, opportunities for further training and/or citizenship [1].

International medical graduates by definition move from the country in which they received their medical training to another where they continue and/or further their training and eventually settle to practise [3]. The terms international medical graduates (IMGs), foreign medical graduates (FMGs) or overseas trained graduates (OTGs) are used interchangeably in academic literature. In this study, the term foreign medical graduates (FMGs) will be used. The US National Library of Medicine refers to an FMG, a MESH term, as physicians who hold degrees from medical schools in countries other than the ones where they practise [4].

South Africa is classified as a middle-income country (MIC) by the World Bank classification of countries according to income [5] and therefore differs from most African countries that are classified as low-income countries (LICs). South Africa, however, in common with many LICs has a critical shortage of medical doctors to serve the health needs of its population [6]. The ratio of doctors is estimated at approximately 2.3 doctors for every 1000 people as compared with an average ratio of 24.8 doctors for every 1000 people in developed countries [7]. This shortage of medical professionals is even more apparent among rural and disadvantaged communities as doctors generally prefer to practise in urban locations.

The term brain drain refers to the transfer of highly educated professionals from low-income and middle-income countries or developing countries to high-income countries or developed countries [8]. The brain drain usually occurs due to a variety of factors like poor working conditions and a lack of job satisfaction in the home country and better educational opportunities and quality of life in host or receiving countries [9]. This transfer of medical professionals worsens the depleted health care human resources of the developing country which usually requires these resources more than the country that is receiving its health care professionals [9].

South Africa is in a unique position in that it is a supplier of human capital; yearly, many South African medics leave to work in HICs like the United Kingdom, Canada and Australia [9] as well as a receiver of human capital in terms of highly skilled health care professionals from various lower middle-income or low-income countries in the world [10, 11].

The health care system in South Africa, thus similarly relies on FMGs to alleviate the shortage of doctors among the rural and disadvantaged communities. Recruitment of foreign health professionals and their employment in South Africa is informed by health and labour legislation and influenced by the World Health Organisations’ (WHO) Global Code of Practice on the International Recruitment of Health Personnel. A 2014 study revealed that foreign medical personnel represent about 1.5% of the qualified public health workforce in South Africa [12]. Graduates from the Southern African Development Community (SADC) countries account for 38% of foreign medical practitioners, specialists, dentists or pharmacists, with 26% from the rest of Africa and 36% originating from the rest of the world [12].

FMGs in South Africa include medical practitioners also from non-SADC African countries, Europe, North America and India. South Africa has agreements with countries including Tunisia, Iran and Cuba for the sourcing of doctors. The agreement with Cuba also includes the training of South African citizens on medical programmes in Cuban facilities [11].

South Africa has had a longstanding and very successful bilateral agreement with the Cuban government [10]. The 1995 intergovernmental agreement facilitated the recruitment of Cuban-origin medical doctors for work in the rural South Africa health sector [10]. This strategy was implemented to address imbalances and unequal health care due to apartheid. The initial agreement was to provide 300 doctors of Cuban origin, i.e. Cuban citizens for primary health care services in rural areas of South Africa [10]. By 1996, the Cuban and South African government entered into a further collaboration, later known as the Nelson Mandela–Fidel Castro exchange programme to train rural and previously disadvantaged South African origin students in Cuban facilities as medical doctors who would commit to practise in the public sector and within mainly rural and disadvantaged communities of South Africa [13].

The deployment of Cuban origin doctors to South Africa has since decreased while the training of South African origin medical students has increased. The intake of students increased exponentially from approximately 96 in 1996, 1200 enrolled in 2013 to more than 3000 students enrolled in 2015 [11].

FMGs who return to practise in South Africa are categorised in one of two groups:

- The first group consists of South African citizens who studied medicine abroad (outside South Africa) and return to practise medicine after qualification. This category includes doctors practising on intergovernmental agreements on the South African-Cuban Medical Collaboration (SACMC). The group also consists of individual South African citizens who pursue private medical studies in countries including for example, Russia, China and Mauritius.
- The second group of FMGs consists of non-South African citizens who studied medicine in their home countries and then immigrated to South Africa to practise medicine. Many professionals in this subgroup are citizens from neighbouring and developing countries who relocated to South Africa for reasons including economic gain, improved training opportunities, citizenship or due to political/religious instability or war in their countries.

This study was conducted to assimilate literature regarding the professional experiences and adjustment of FMGs to host country environments in middle-income and developing countries similar to South Africa. The information gained through this process will help medical educators and health providers to support and assist FMGs who work in local South African settings as well as provide insight for health care educators working in other similar MICs.

The specific objectives of the study were to:

1. Explore the professional experiences of FMGs upon entering host countries similar to South Africa.
2. Identify factors that facilitate the adjustment of FMGs upon entering the country of practice.

## Methods

The five-step methodological framework of Arksey and O’Malley was used to guide the review [14]. These steps included the following:

1. Identifying the research question(s);
2. Identifying the relevant studies;
3. Study selection;
4. Charting the data;
5. Collating, summarising, and reporting the data;

1. Identifying the research question(s)

The research question was to identify academic published literature regarding the professional experiences and adjustment of FMGs to host country environments in middle-income countries.

The research questions were intentionally a broad one in keeping with the view that scoping review design represents a methodology that allows assessment of emerging evidence as well as representing the first step in a larger project or research development [15].

2. Identifying the relevant studies

A literature review was conducted using the databases MEDLINE and PUBMED to identify articles documenting the objectives as stated above. An initial search was conducted using relevant keywords, including “FMG”, “foreign medical graduates”, “experiences”, “adjustment”, “adaptation”, “integration”, “acculturation” and “assimilation”. The keyword FMG was used alone as well as in combination with all the other keywords as noted above.

The term “adjustment” involves a process to modify one’s behaviour in changed circumstances and refers to the ability to acclimatise and conform to new conditions to fulfil psychological, physiological and social needs [16]. Synonyms for adjustment include assimilation, integration, acculturation and adaptation, and these were therefore also used as search terms.

The inclusion and exclusion criteria were discussed and agreed upon by the two reviewers, and consensus was reached about the inclusion of the final number of articles (refer to Table 1).

**Table 1:** Exclusion and inclusion criteria

Criterion	Inclusion criteria	Exclusion criteria
Study focus	International medical graduates- medical doctors	Nurses or other health care professionals
	Foreign medical graduates - medical doctors	Residency matching of FMGs in the USA and Canada
	Overseas trained graduates - medical doctors	Psychiatric residencies
		Papers that focused specifically on refugees
		Non-English articles
Type of article	Original research	Letters, commentaries, editorials
		Grey literature
		Programme evaluations
		Systematic reviews
Time period	Between 1 January 2000 and 31 December 2016	Before 1 January 2000 and after 31 December 2016
Location	Rural or urban	
Type of doctor	General/primary care/rural	Psychiatrists in training

The inclusion criteria to select articles for the review included articles published between 1 January 2000 and 31 December 2016. Inclusion criteria included publications that addressed experiences of FMGs while working in clinical practice.

Exclusion criteria involved articles not written or available in the English language or those articles outside of the specified period were excluded. Papers that were specific to nurses and other health care professionals were excluded as the review focused on medical doctors or physicians specifically. In addition, articles that specifically focused on experiences of FMGs in residencies or experiences with residency application and residency matching for the United States of America and Canada were excluded as similar programmes do not exist within middle-income countries like South Africa and it was considered that it was outside the research question. Commentaries, editorials, organisational reports, grey literature, programme evaluations and letters were excluded. Review articles were excluded as the emphasis was to get to the primary data sources rather than secondary sources in systematic or scoping reviews. Papers that focused specifically on refugees were also excluded as these doctors were considered to be a very special subset of doctors whose experiences were determined to a large extent by the trauma suffered from leaving their home countries. Publications that met the inclusion criteria were sought and included.

3. Study selection

**Fig. 1:**
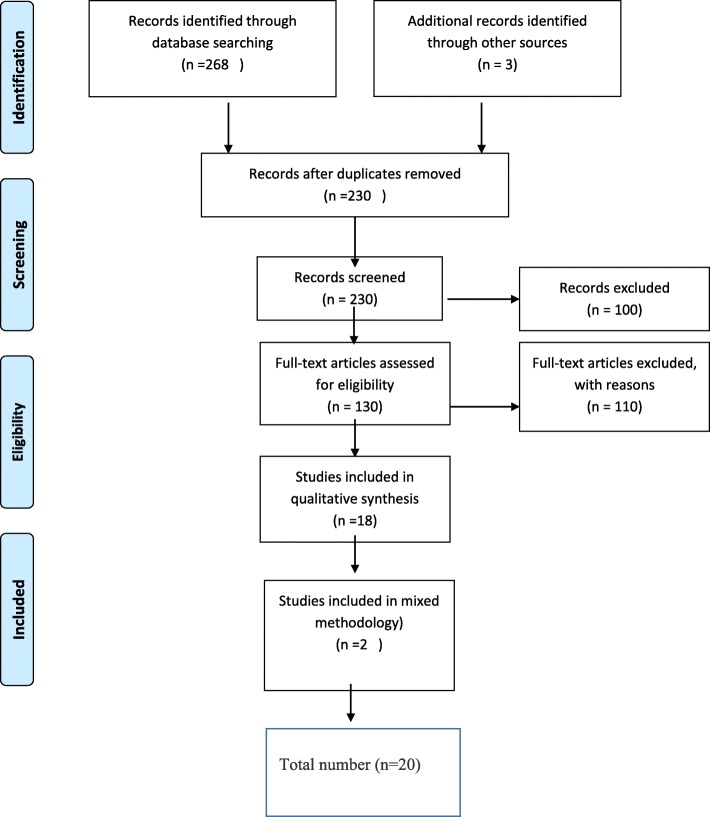
Process of the literature search

A systematic process was used to select the literature for the scoping review. Firstly, the results of the searches were imported into a reference management programme. The records identified through database searching were 268. An additional three records were identified through other sources. Of the total 271 records, 41 duplicate records were identified and discarded leaving 230 records. These 230 records were screened by two researchers independently. After screening, the 230 abstracts to determine their relevance to the review purpose and the objectives, 100 articles were deemed out of the scope of the review and were subsequently discarded. Finally, 130 full-text articles were assessed for eligibility. Thereafter, 110 full-text articles were excluded with reasons. The final number of studies that were included was 20 (refer to Fig. 1 and Table 1).

4. Charting the data

The categories for data extraction reflected our research questions. The outputs were plotted on data extractor sheets for thematic analysis to be made. The themes that emerged most frequently were extracted by the two reviewers. The themes were developed after each researcher carefully read and reread the literature. Four major themes emerged from the data, and minor themes were developed inductively and used to organise the information in each of the four themes, as described and shown in Table 2.

5. Collating, summarising and reporting the data

**Table 2:**
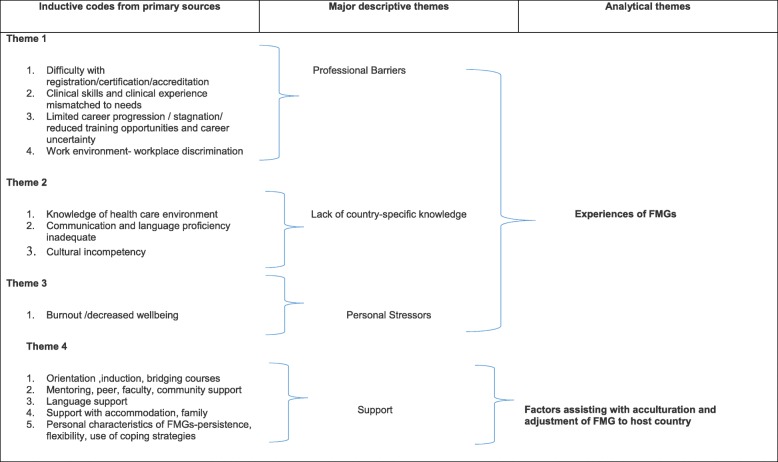
Evidence of process of thematic analysis

The data were collated into qualitative thematic summaries, and as most of the data was qualitative in nature and was summarised into narrative synthesis. The findings were analysed in relation to the research question and the objectives of the scoping review. Gaps in the literature were identified.

Ethical approval for the study was obtained from the University of KwaZulu-Natal: Humanities and Social Sciences Research Ethics Committee.

Figure 1 provides an overview of the process followed and literature identified during the data collection process. The initial search for published articles on the experiences of FMGs between 1 January 2000 and 31 December 2016 yielded 271 publications, which were reduced to 20 after the removal of duplicates, the removal of ineligible articles and the application of the exclusion criteria. Eighteen of the 20 studies that were deemed relevant to the experiences of FMGs were conducted by studies that followed a qualitative research design and two were conducted using a mixed methodology design (refer to Fig. 1).

## Results

In response to the objective that explored the scope of publications on the experiences of FMGs, the articles in this review mainly describe research studies that were conducted in various HICs. A total of six studies had been conducted in Canada, one of which included a combination of Canadian and Swedish research; four in Australia; two each in the United Kingdom and Finland; then one study each from New Zealand, the United States of America, Ireland, Germany, Austria and the Netherlands. Publications reviewed within the search period included the earliest publication in 2004 and the most recent in 2016. Of particular interest was the absence of publications from Africa and South Africa.

In answer to the objective that set out to describe the types of challenges reported by FMGs, three themes were found to be common as indicated in Table 2. The frequency of these descriptions suggests some degree of consensus that firstly confirm that FMGs experience a degree of difficulty in new host countries and the literature also provided insight into factors that could assist in the adjustment of FMGs to their new work countries.

FMGs reported professional barriers, lack of country-specific knowledge and stress as the most common sources of barriers on entering host countries while personal and professional support and the personal characteristics were some of the factors reported that facilitated their adjustment to the host country.

Although there are differences in the size of FMG populations in various HICs, they do exhibit evidence of extensive reliance on the use of FMGs in their overall health care and dependence on human resources [17].

### Professional barriers

Studies revealed that FMGs experienced various barriers to registration, licensure and in navigation accessing correct information within the health system [18–22]. In some cases, these barriers resulted in an inability to work in their profession, while other FMGs found work in related fields [19].

In the context of policy regarding FMGs in Canada, Borgeault and Neiterman noted several barriers for internationally trained health professionals. These include poor information to prospective FMGs, a lack of transparency about how to register and difficulty in having the educational credentials of FMGs recognised [17].

In the German context, FMGs similarly criticised the registration process for being slow, confusing and bureaucratic. The application to register for a specific hospital position was also criticised as physicians felt that their future employer had lied to them about working conditions at the hospital [20].

Among the many barriers reported to accreditation and registration to work in Austria where the requirement for many FMGs to repeat their internship or even their whole education, which set them back several years. The training in Austria was particularly challenging as FMGs had to master a language, which further complicates the situation [19].

While the barriers to licensure, accreditation and registration predominated in literature from Anglophone countries including Canada, Australia, the United States of America, New Zealand and Ireland, it featured as an even more complex factor in the non-English-speaking countries such as Germany, Austria and the Netherlands.

The same issues are reported in Finland. One of the major concerns, especially among physicians trained outside the EU/EEA trying to enter the profession in Finland, was access to work in the health care sector made problematic by a difficult licencing process, lack of information, bureaucratic difficulties and what FMGs experience as unfair test requirements [21].

In a New Zealand study, FMGs noted a lack of information about the requirements to pass the exams [23]. FMGs perceived an overall lack of information, a lack of information specific to the New Zealand health system and limited places from where to access information throughout the process of registration also in finding employment and integrating into the workforce [23].

FMGs in host countries also experienced barriers in the practice of their professional and clinical skills [22, 24–29]. They reportedly required teaching on how to interact within the health care system and opportunities to practise specific professional and clinical skills for effective practice in the new settings [24]. In the German context, FMGs reported feeling as if they lacked certain clinical competencies which they considered as necessary for successful clinical interactions such as the necessary experience with treating certain diseases, for example, tuberculosis (TB) because certain diseases were not prevalent in their country of origin [20]. FMGs reported similar problems in using certain diagnostic/therapeutic tools and expressed that they felt that they required competencies in Germany exceeding their level of specialisation.

FMGs in Germany indicated their ability to speak in German as a significant barrier as they struggled with various aspects of language, difficulty in understanding the general everyday language, understanding unfamiliar medical terminology and the use of colloquial terminology for various diseases and medical conditions [20].

### Limited career progression

FMGs in the United States of America reported various barriers to professional opportunities, limitations in practice location, choice of work, field of speciality and opportunities for advancement within various fields. They, however, recognised the professional limitations as part of the trade-off upon accepting work in the United States of America [30]. In this way, the FMGs to the United States of America, as opposed to FMGs from Ireland and the United Kingdom, viewed the barriers and limitations as part of the “transactional cost of living and working in the United States of America” and they still perceived their professional experiences in the United States of America as significantly greater than those in their home countries [30].

FMGs recruited to Ireland experienced a de-skilling process. This was especially noticeable, among doctors from non-European Union countries. They encountered limited training opportunities; stalled career progression within the Irish health system in a process described a “brain waste” [27]. The Irish health system also relied more on services rendered by junior hospital doctors, and vacancies were often at the junior hospital doctor level. In this way, FMGs to Ireland missed out on formal postgraduate training schemes and were offered only limited opportunities for career progression [27].

Austrian FMGs similarly reported not being able to work in their chosen environment and experiences of potential not fully being realised. The inability to work in their chosen health profession often resulted in frustration for FMGs who felt an inability to continue in their professions their professional knowledge being undervalued [19].

Despite having satisfied the entry requirements, FMGs in the New Zealand context of many FMGs who struggled to find employment and to integrate into their employment role or even to find medical employment [23]. FMGs to New Zealand reported significant delays between passing their exams and receiving a job offer. This meant that those who were unable to find work either had to move out of the country or were forced to considered work outside the medical profession.

### Work environment and workplace discrimination

FMGs experienced both overt and subtle forms of workplace bias and discrimination which occurred at all levels of the workplace and in interaction with patients, colleagues and with their supervisors. They also noted less overt examples at institutional leadership level. Chen described workplace bias and discrimination as occurring at a systematic level [30].

In this way, FMGs, irrespective of the number of years in US practice, perceived themselves as being held to a different standard of practice than their US-trained counterparts [30].

FMGs describe challenges in the transition to the culture and practice of medicine in the United States of America and normative work-related procedures, such as interviewing for residency [30].

Although FMGs from countries in the Middle East reported being asked discriminatory questions during job interviews, religion and appearance were not reported as real barriers in most of the studies. In a study conducted in Germany, male doctors from the Middle East had reported considerable difficulty working with the medical team during the initial period of practice. These FMGs reported feeling being discriminated by doctors and nurses but their experiences improved with time and as mutual respect and trust developed within the team [18].

FMGs in Germany experienced rejection and discrimination which they frequently attributed to their status as being a “foreigner”. They reported difficulties in interpersonal interactions with patients, colleagues and superiors and felt badly treated by colleagues (including nurses) and patients [20].

### Lack of country-specific knowledge

With reference to the lack of country-specific knowledge of FMGs, the findings revealed that their knowledge of the health system, clinical skills and disease profiles did have an impact on their ability to adjust into the health care environment of the new country. FMGs in the various countries displayed variability in the medical knowledge, clinical skills and professional attitudes due to the variability of their undergraduate training and the various processes taught at undergraduate level to integrate their knowledge and clinical reasoning [20, 23, 25, 31].

FMGs in many of the studies and countries reported a mismatch between the tasks assigned to them and their level of expertise. They reported a lack of knowledge regarding their roles in the context of medicine of the new country [20]. FMGs across the majority of countries experienced that they were frequently placed and employed in positions of greatest medical need, but that these needs had been inadequately matched to their clinical expertise and their previous experiences in the country from where they had graduated [26, 27].

Areas of greatest medical need often have greater working challenges and are often understaffed as the local indigenous/native professionals of the host country also find these positions challenging and hence refrain from accepting positions to practise in these areas, e.g. in Ireland. FMGs are recruited to work in posts with working conditions unacceptable to Irish-trained doctors [27]. These conditions may include areas that are very rural, often primary health care that offers limited services for the community, with a high burden of disease and very vulnerable populations [25–27].

FMGs in Australia reported professional isolation, faced with a heavy work load and expectations of a high level of medical care despite their often inadequate skills for rural practice, lack of access to specialists and having to move frequently for different training opportunities [25].

### Cultural competence

Research with FMGs identified various challenges both in their language proficiency and in the process of cultural transition. They reported cultural barriers and a lack of awareness of cultural norms in caring for patients from diverse cultures [20, 23, 25, 26, 30, 32, 33], and difficulty across linguistic barriers in professional and personal communication in interactions with patients and colleagues [13–15, 20, 21, 28, 29, 34]. Language and cultural barriers were more often reported where FMGs migrated to non-English speaking countries, such as to the Netherlands, where those without a good command of the Dutch language experienced significant language barriers [18].

FMGs to English-speaking countries also reported difficulties in knowledge of the English language but also with specific difficulties in understanding subtle aspects of language such as used in sarcasm and colloquialisms. They report missing out on non-verbal communication, non-verbal clues and missing out on facial expression and various uses of body language [18, 21, 28, 29, 33]. Participants in Chen et al.’s study reported similar difficulties of subtle aspects of language, such as use of colloquialisms, sarcasm and idioms [30].

Participants perceived a loss of autonomy as physicians in the United States of America, with its emphasis on shared decision-making, in contrast to experiences in their home countries. This loss of autonomy sometimes led to decreased confidence. Finally, respondents were unaccustomed to the system of checks and balances in US health care and physicians’ sensitivity to potential litigation [30].

In the Netherlands, FMGs expressed frustration with the written examination being administered in the Dutch language at Maastricht University. They thought that the examination should be preceded by training in Dutch medical terminology. Language problems were considered a significant barrier during and after, study. Bad experiences relating to language, have reportedly led to FMGs ultimate rejection of a medical position [18].

FMGs to Finland described the Finnish language as difficult to learn, language courses in short supply and or of poor quality. FMGs also perceived that the Finnish system had failed to support them in language training, and their lack of language skills prevented FMGs from entering the Finnish system [21].

Understanding patient-centred communication is a major challenge for FMGs in their integration in the Australian health system. This difficulty is often a major shift from the culture in the country of origin of the FMG. While FMGs traditionally study in systems with a paternalistic doctor-dominated communication system, they experienced this very different to the Australia setting where the more educated and informed consumers demanded higher levels of information and discussion [35].

Organisational support, such as professional and cultural mentoring, were commonly identified needs of FMGs in Australia. Relationships were strengthened when staff and FMGs met socially and discussed cross-cultural issues, thereby establishing effective relationships within the Indigenous community [32].

### Stress

Studies showed that FMGs to the majority of the countries represented in this review experienced professional and personal stress due to lack of personal and professional support [20–24, 26, 28, 29, 34]. Stress presented in the forms of workplace discrimination and bias at various levels of interaction with patients, nurses and colleagues [18–20, 25, 30], lack of choice regarding working hours, type of work, career opportunities and career progression [25]. They also faced constant incidences of career uncertainty [21, 23, 33], felt held to different standards in the practice environments from their locally trained counterparts and needed more time to transition into new roles [18, 19, 21–26, 28–30, 33, 34].

A particular study from Finland had reported that FMGs faced a high risk of burnout and poorer work ability due to increased stress which ultimately resulted in FMGs having lower occupational well-being as compared with their locally trained counterparts [36].

### Support: professional and personal

In response to the objective that explored the factors that were reportedly useful for FMGS to adjust to the new settings; the most comprehensive reports suggest for effective and comprehensive orientation. This was deemed essential to facilitate the acquisition of knowledge and to adjust to a new working environment [4, 7, 10, 12, 13, 22, 33, 34], as well as facilitating the sociocultural connection within the community which was identified as an important factor in fostering integration [22, 26, 28, 29, 32, 33].

The orientation sessions reported were either offered in the form of an induction or a bridging programme [32]. Support from faculty mentors and peers greatly facilitated the acculturation process for FMGs [22]. Both personal and professional support to FMGs along the journey and the use of mentoring were reported strategies that facilitated adjustment [18, 22, 24–26, 28, 29, 32, 33]. Han and Humphreys’ study in Australia showed that professional support from professional organisations and agencies combined with support from colleagues and supervisors, contributed significantly to increased professional satisfaction in a rural setting [25].

Information about banking, housing, schooling and recreational opportunities was identified as an important need for new IMGs. Support for the spouses of FMGs was also noted as an important component of orientation. Facilitating sociocultural connection within the community and with others from the same cultural background was also identified as an important factor in fostering integration into community and place [26].

Family support, hospital provided accommodation and administrative assistance from recruitment agencies were helpful to FMGs to settle into a new country and facilitated the adjustment process [26, 33].

Communication and language support were also a significant factor in the adjustment process of FMG resocialisation [18, 32–34]. The first few months following the point of entry into a medical position are a crucial time for the majority of FMGs in experiencing difficulties with communication. The importance of speech and language skills and the serious implications thereof for clinical practice of FMGs were reported across English-speaking and non-English-speaking countries [18, 32–34]. There is a great need to contextualise the learning of language strategies with staff within existing frameworks commonly used to improve the communications skills [34]. FMGs who had taken additional language courses were able to improve their communication with patients and colleagues [18, 32] and found that knowledge of indigenous languages and understanding of accents and different cultural norms had helped them with successful integration.

Facilitating factors also included personal characteristics and strategies that many FMGs have characteristics including persistence, flexibility knowledge and experience gained from their country of origin. These had helped them to be successful in the host country in spite of multiple problems [18, 32]. FMGs also used various strategies to integrate and feel a sense of belonging in the host country. These included emphasising the similarities that they share with locally trained doctors or using their professional status as internationally trained medical graduates [37]. Some of the FMGs have had to adjust their professional image to fit into the health system and many do this by minimising the differences between professional practice in the host country versus their country of origin or by asserting the superiority of the professional approaches acquired in their countries of origin [28]. Other strategies like maintaining an optimistic attitude, reframing their experiences in a more positive light and trying to blend in helped the FMGs to integrate and adapt to the host country [22].

FMGs also felt positively about their unique skills and advantages that they brought to the host country. Many viewed aspects of their prior training and clinical practice as professional assets, identifying skills and advantages gained through experience in another health care system and sociocultural context. Many FMGs felt that theiroutsider status allowed them to better empathise with patients from ethnic/racial minority groups [30].

## Discussion

This study assimilated publications on the experiences of FMGs and the reported factors that facilitate their adjustment to new countries. The purpose of our scoping review was to explore available published academic literature on this subject. The search included published material for the period January 2000 to December 2016. While the literature on issues relating to FMGs was abundant, the literature regarding the specific aims of the study was not as prolific. There was a distinct absence of literature on FMGs from the African continent and from middle- and low-income countries. We found no reports of the experiences of FMGs and their adjustment to settings similar to South Africa. This result was surprising given that the foreign medical personnel represents about 1.5% of the qualified South Africa public health workforce [1].

The predominance of literature from HICs including Canada and Australia could be indicative of the historical recruitment of doctors from L- and MICs to HICs. Research on this phenomenon is therefore more readily available from HICs who serve as the major recipients of FMGs. The term “brain drain” has furthermore been associated with the movement of educated, talented professionals from LICs to HICs. Low and middle-income countries, including South Africa, generally do not have a tradition of recruiting FMGs. As evidenced in our yield, there is also very little research on this topic from donating countries.

The results show that HICs do rely on FMGs to supplement their workforce and therefore more literature and research emanates from these recruiting countries. Eighteen of the studies were qualitative and descriptive in nature, were based on relatively small participant samples and hence restrict the generalisability of the findings.

The competencies of FMGs have received much media and political attention in the countries that regularly receive large numbers of FMGs. In those settings, the training programmes focussed to up-skill and address the orientation needs of FMGs [2].

Some countries have high litigation sensitivity among their relatively more educated populations. In those countries FMGs’ challenges included adjustment, which have had an impact on patient care has been highlighted with cases of litigation [2]. This has also led to various governmental and institutional policies and recommendations with regard to supporting such individuals who are recruited into the country to avoid any medico-legal issues. The process of active recruitment also facilitates the possibility of screening to assess the competency of doctors as opposed to allowing doctors to migrate or emigrate for economic reasons of their own accord. The results indicate that FMGs who migrate to HICs generally faced similar experiences and reported similar challenges to integration and adjustment.

FMGs experienced various barriers to registration, accreditation and licensure in the majority of the HICs. The high predominance of this in the literature came as no surprise as the highly regulated environment in HICs was noted to add to the difficulty in making entry and adjustment of FMGs. This had the effect of delaying employment, FMGs working outside their chosen field and a resultant mismatching and underutilization of FMGs which contributes to the brain waste that occurs in some HICs.

FMGs experienced both overt and subtle forms of workplace bias and discrimination. While some discrimination was reported on as foreigners in a new country, it was difficult to determine the significance of this and the prevalence of it as the results presented within most papers did not reflect the significance of gender or religious bias on FMG experiences. Only one German study mentioned qualitative results of the impact of religion and gender on a small sample size.

FMGs in many of the studies and countries reported a mismatch between the tasks assigned to them and their level of expertise. They reported a lack of knowledge regarding their roles in the context of medicine of the new country. They were frequently placed and employed in positions of greatest medical need with greater working challenges.

Language and culture played an important role in the FMG experience. While this was more significant for non-English-speaking countries like Austria, Finland and Germany, it was also reported in English-speaking countries where FMGs struggled with the nuances of language acquisition and understanding in medical contexts and the use of colloquial terms for medical conditions. Also, entrance exams in foreign languages was difficult for FMGs and an additional barrier.

The factors that were reportedly useful for FMGS to adjust and integrate to the new settings globally were induction, orientation and bridging programmes which played a major role in providing support for FMGs to integrate into their new host countries [38].

Communication and language support were also a significant factor in the adjustment process of FMG resocialisation. Communication was found to be essential in the workplace with colleagues and peers and effective communication was essential to facilitate the acquisition of new knowledge.

### Implications of findings for MICs and LICs

Unfortunately, due to the lack of any literature from South Africa, MICs and other developing countries, it was not possible to make direct comparisons between conditions in South Africa and other developing countries.

Regarding the regulatory frameworks and policy governing entry and practice of FMGs in HICs, it is obvious that HICs have more defined policies and regulatory bodies to regulate licensure and registration than MICs and LICs.

FMGs in HICs found the registration, accreditation and licensure process a frustrating process and hence a significant barrier on entry. The regulatory bodies of HICs are better developed than MICs and LICs, and as FMGs still found them to be barriers to registration and licensure, we would surmise that this problem would be even more frustrating for FMGs trying to enter countries like South Africa. Requirements to register with the local regulatory body, the Health Professions Association of South Africa, the HPCSA, are not a transparent or easy process for foreign nationals either. Licensure in South Africa requires the passing of a foreign graduate exam with the major language medium of examination being in English which is a barrier for FMGs from Cuba and other foreign countries.

Cuban trained medics on entering South Africa struggle with language and understanding local terminology have difficulty with clinical skills and lack the knowledge of disease profiles locally as well as understanding our health care system [3]. Hence, professional barriers to licensure, registration and accreditation would be even more of a challenge in MICs and LICs. South Africa has 11 official languages and a variety of cultures within its population which adds to the complexity of clinical practice for FMGs.

Rural environments have higher disease burdens than urban areas and lack infrastructure, medical resources like equipment and drugs to adequately offer good medical services in certain areas which add to the stress and burn out that FMGs would be likely to suffer in MICs and LICs even more so than that described by FMGs in HICs. FMGs find themselves as not being adequately skilled for the local context. Similarly, FMGs from other countries in Africa are unfamiliar with South African indigenous traditions and cultures and therefore are ostracised for not being familiar with local culture and practice. Bias and xenophobia have played out in rural areas of South Africa. Finally, in terms of support required to integrate FMGs into a country, MICs have a dual role of accrediting body as well as gatekeeper and the bureaucracy around how to support FMGs in South Africa is still in the infancy stage. Local efforts are being made to run induction, bridging and orientation programmes but they are noted to be few and far between. While communication training in indigenous languages is now being recognised as essential for undergraduate training, this has not yet been offered to FMGs entering South Africa.

### Limitations of the study

The results reported above are subject to certain limitations. The articles reviewed were limited to HICs as the searches revealed no local literature from Africa or South Africa, and experiences and factors may therefore only be representative of the local, regional or national experience of those countries. Placing strict restriction on the various inclusion and exclusion criteria at the start of the study was necessary to focus this review; however, that means that there is a possibility of publication bias. For example, only English-language sources were retrieved and reviewed. No doubt, there is literature on FMGs that has been produced in other languages. Another limitation of this review is that its scope may not be broad enough because only scientific papers were included. Additionally, because of our narrow search string, we may have missed some relevant papers on the subject. Finally, a scoping review cannot present absolute truths, because we did not conduct a quality assessment of reviewed sources. The results should therefore be interpreted with some caution.

## Conclusions

The scoping review revealed a lack of published literature on the experiences of FMGs in Africa and specifically in South Africa and all the studies included in this review were conducted in HICs. Our findings also revealed that FMGs generally experience both professional and personal challenges which can successfully be addressed through comprehensive orientation and appropriate support. There is a significant lack of literature about FMGs.

This scoping review provided sufficient evidence of challenges including in the workplace environment that impact on the well-being and integration of the FMGs to the host country; however, what is not known about FMGs from the literature reviewed is what happens to those who fail to adjust and integrate themselves into the host country, i.e. whether they experience higher litigation from their patients, what career pathways they ultimately choose, whether they contribute positively to the country in other ways or whether they leave medicine due to the challenges experienced and go on to other careers (Table 3).

**Table 3:** Summary of published articles

Title/reference	Design	Location	Aims and objectives	Issues reported
Employment, psychosocial work environment and well-being among migrant and native physicians in Finnish health care [36].	Mixed method study	Finland	The study focuses on integration of migrant physicians into Finnish health care by comparing their employment situation, perceptions of work-related stressors and well-being with the experiences of native physicians in a large representative survey study.	Migrant physicians are more likely to be employed in primary care and work more often on-call than native Finns. Lack of professional support burdened migrant physicians more than the native Finns, while stress related to poorly functioning information systems was reported more often by native physicians. Migrant physicians evaluated the management in health care organisations as more fair, but they reported more work-related distress compared with native Finns.
Integrating international medical graduates: The Canadian approach to the brain waste problem. Wanted and Welcome? [17]	Qualitative	Canada	This chapter describes the Canadian approach to the brain waste problem associated with IMGs and maps out some of the recent efforts to address the problem of “brain waste” in the Canadian medical sector with a specific focus on the province of Ontario.	The chapter maps the recent efforts to address the problem of Brain waste in the Canadian medical sector. An increasing number of policies that have been developed to assist in integrating IMGs into the Canadian labour market. Many of the difficulties IMGs confront in trying to enter the Canadian health care system come as a result of the lack of coordination, not only between the provincial and federal governments but also between different stakeholders with very different mandates vis-à-vis IMGs, therefore solutions to these problems require coordinated approaches. As other countries move toward a Canadian-style immigration system, the difficulties faced by IMGs in Canada may serve as a cautionary tale. All countries should have in place more formal mechanism for facilitating the integration of highly skilled immigrants into their labour markets so as to avoid (as much as possible) brain waste problems.
Professional experiences of international medical graduates practising primary care in the United States [30].	Qualitative	USA	To characterise the professional experiences of non-US born IMGs from limited-resource nations practising primary care in the USA.	Four recurrent themes: IMGs experience overt and subtle forms of workplace bias and discrimination; IMGs recognise professional limitations as part of “the deal”; IMGs describe challenges in the transition to the culture and practice of medicine in the USA; IMGs bring unique skills and advantages to the workplace.
A qualitative study of the international medical graduate and the orientation process [26].	Qualitative	Canada	The purpose of this qualitative study was to explore perceptions of, and experiences with, orientation processes for new IMGs.	New IMGs need to learn about the health care system and the peculiarities of the specific practice context in which they will be working. Orientation needs to include opportunities for reflecting on one’s own cultural biases and for learning about the cultural background and beliefs of a new patient population. Mentoring and effective integration within the community also emerged as important components of effective orientation processes
“If it wasn’t for OTDs, there would be no AMS”: overseas-trained doctors working in rural and remote Aboriginal health settings [32].	Qualitative	Australia	This paper focuses on recent research carried out in Australia to analyse factors affecting OTS’s professional, cultural and social integration and examine their training and support needs.	The need to better address recruitment, orientation and cross-cultural issues; the importance of effective communication and building community and institutional relationships, both with the local health service and the broader medical establishment.
Communication skills, cultural challenges and individual support: challenges of international Fmedical graduates in a Canadian health care environment [24].	Qualitative	Canada	Conducted a needs assessment to assess Canadian IMGs communication skills needs.	IMGs required a combination of language skills, teaching on how to get things done in the health care system, opportunities to practise specific skills, support systems, faculty and staff education in relation to cultural challenges.
Overseas-trained doctors in Australia: community integration and their intention to stay in a rural community [25].	Qualitative	Australia	The aim of this study was to identify the factors that influence foreign doctors’ community integration and examine how these affect their intention to stay in the rural community.	Maintaining cultural and religious values, as well as relationships to their respective ethnic communities is important to OTDs. While they do not expect excessive support from the community they appreciated the cultures of welcoming or “embracing differences”. Supportive communication and supervisory support positively influence OTDs’ appreciation of what the rural community can offer them and how they might overcome any difficulties that they face with their rural practice and life.
Barriers and facilitating factors in the professional careers of international medical graduates [18].	Qualitative	Netherlands	This article concerns IMGs who enter the Netherlands and as their non-European medical qualifications are not considered equivalent to the Dutch qualifications, they are required to undertake additional medical training. Because little is known about their professional careers, we set out to identify the barriers that confront and the facilitating factors that support IMGs before, during and after their supplementary medical training	Difficulties were reported in accessing information on complementary medical education and lack of (financial) support. Perseverance was reported to be essential. Financial and social support were also reported as facilitating factors. Lack of command of the Dutch language and age were seen as barriers to securing employment and entrance to specialisation
A cycle of brain gain, waste and drain-a qualitative study of non-EU migrant doctors in Ireland [27].	Qualitative	Ireland	This paper provides insight into the experiences of non-EU migrant doctors in the Irish health workforce.	Respondents believed they had been recruited to fill junior hospital doctor “service” posts. These posts are unpopular with locally trained doctors due to the limited career progression they provide. Respondents felt that their hopes for career progression and postgraduate training in Ireland had gone unrealised and that they were becoming de-skilled. As a result, most respondents were actively considering onward migration from Ireland.
“Why should I have come here?”-a qualitative investigation of migration reasons and experiences of health workers from sub-Saharan Africa in Austria [19].	Qualitative	Austria	The objective was to explore foreign medical graduates’ reasons for migration to Austria, as well as their personal experiences concerning the living and work situation in Austria.	For most participants, the decision to migrate was not professional but situation dependent. Austria was not their first choice as destination country. Several study participants left their countries to improve their overall work situation. The main motivation for migrating to Austria was partnership with an Austrian citizen. Other immigrants were refugees. Most of the immigrants found the accreditation process to work as a health professional to be difficult and hindering. This resulted in some participants not being able to work in their profession, while others were successful in their profession or in related fields. Participants report experiences of discrimination, but also positive support.
Difficulties experienced by migrant physicians working in German hospitals: a qualitative interview study [20].	Qualitative	Germany	This study provided an overview of the multifaceted difficulties migrant physicians face in German hospitals.	Participants described difficulties relating to health care institutions, own competencies, and interpersonal interactions. Participants experienced certain legal norms, the regulation of licensure and application for work, and the organisation of the hospital environment as inadequate. Most struggled with their lack of setting-specific (language, cultural, clinical, and system) knowledge. Furthermore, behaviour of patients and co-workers was perceived as discriminating or inadequate.
Inflows of foreign-born physicians and their access to employment and work experiences in health care in Finland: qualitative and quantitative study [21].	Mixed methodology	Finland	This study (i) examined the numbers of foreign-born physicians migrating to Finland and their employment sector, (ii) examined, based on qualitative interviews, the foreign-born GPs’ experiences of accessing employment and work in primary care in Finland and (iii) compared experiences based on a survey of the psychosocial work environment among foreign-born physicians working in different health sectors (primary care, hospitals and private sectors).	The number of foreign-born physicians has increased dramatically in Finland since the year 2000. In 2000, a total of 980 foreign-born physicians held a Finnish licence and lived in Finland, accounting for less than 4% of the total number of practising physicians. In 2009, their proportion of all physicians was 8%, and a total of 1 750 foreign-born practising physicians held a Finnish licence and lived in Finland. Non-EU/EEA physicians experienced the difficult licencing process as the main obstacle to access work as a physician. Most licenced foreign-born physicians worked in specialist care. Half of the foreign-born GPs could be classified as having an “active” job profile (high job demands and high levels of job control combined) according to Karasek’s demand-control model. In qualitative interviews, work in the Finnish primary health centres was described as multifaceted and challenging, but also stressful.
Perceptions of migrant doctors joining the New Zealand medical workforce [23].	Qualitative	New Zealand	This study identified and explored issues of concern to OTDs when first integrating into the New Zealand medical system through the New Zealand Registration Examination (NZREX) pathway.	Work issues which included difficulty finding employment and difficulty integrating into their work role; a bridging programme which improved the ability of OTDs to gain knowledge and experience of the New Zealand medical working environment; financial difficulties included a major impediment to attaining registration and a career pathway in New Zealand; and bureaucratic barriers (including examinations and information availability), which were seen as necessary but unsympathetic processes in gaining registration
Doctor-patient communication issues for international medical graduates: research findings from Australia [35].	Qualitative	Australia	This study presented a sub-set of findings on the factors associated with speech and language practices for IMGs, taken from a qualitative study which examined the IMGs’ experience of integration into the Australian health care system.	Findings indicated that the months following the point of entry into a medical position were a critical time for the majority of IMGs in terms of difficulties with communicating in English. The findings emphasise the importance of speech and language skills and the serious implications of this issue for the clinical practice of IMGs.
Liaison Officer for International Medical Graduates: Research Findings from Australia [33].	Qualitative	Australia	This article presents findings from Australian-based research which explores the IMGs’ experience during entry to their chosen country and posits the need for a designated liaison officer to help support the transition.	The findings document factors associated with the decision to leave their country of origin, psycho-social aspects of stress experienced upon arrival in Australia, and the participants’ perspective on the suggestion for the appointment of a hospital-based liaison officer to assist IMGs during the transition process
Navigating otherness and belonging: A comparative case study of IMGs’ professional integration in Canada and Sweden [28].	Qualitative	Canada and Sweden	This paper explores the *othering* processes and feelings of *belonging* among international medical graduates (IMGs) who seek to practise medicine in Canada and Sweden. Building on the theoretical literature on *othering*, *belonging,* and the conceptualisation of status dilemmas, they explore how IMGs in Canada and Sweden negotiate their professional identity, how they cope with being *othered* and how they establish a path to *belonging*.	Feelings of belonging to a professional group Canadian or Swedish do not seem to be static but rather fluid ephemeral and changing depending on the context. They demonstrate that the construction of professional identity among IMGs necessitates constant comparison between the differences and similarities among “us”—immigrant physicians, and “them”—local doctors. In this process, one’s ethnicity, gender, and professional status are intertwined with the experience of being seen as “the Other”. They also show that in negotiating their professional status, IMGs actively interpret the meaning of being a Canadian/Swedish physician. They conclude that feelings of belonging to a professional group (Canadian or Swedish) do not seem to be static but rather fluid, ephemeral and changing, depending on the context. Our analysis suggests that more attention should be paid to the social context in which experiences of processes of being *othered* and feeling *belonging* are being constructed and interpreted by people themselves
Professional integration as a process of professional resocialization: Internationally educated health professionals in Canada [28].	Qualitative	Canada	The study examined the process of professional resocialization among internationally educated health care professionals (IEHPs) in Canada	During professional integration, internationally educated health care providers modify their approach to professional work. This process can be conceptualised as professional resocialization. While some aspects of professional identity are modified, others persist. Professional resocialisation takes time. While the reliance on international health care providers will most likely remain a feature of the majority of health care systems of the developed world, various stakeholders need to recognise the cultural specificity of professional practice in the international health care providers’ integration process.
Experiences of non-UK-qualified doctors working within the UK regulatory framework: a qualitative study [29].	Qualitative	United Kingdom	This study explored the experience of non-United Kingdom-qualified doctors in working within the regulatory framework of the General Medical Council (GMC) document for Good Medical Practice	Information and support for non-United Kingdom qualified doctors has little reference to the ethical and professional standards required of doctors working in the United Kingdom. Recognition of the ethical, legal and cultural context of United Kingdom health care occurs once doctors are working in practice. Non-United Kingdom qualified doctors reported clear differences in the ethical and legal framework for practising medicine between the United Kingdom and their country of qualification, particularly in the model of the doctor-patient relationship. The degree of support for non-United Kingdom-qualified doctors in dealing with ethical concerns is related to the type of post they work in.
“That’s your patient. There’s your ventilator”: exploring induction to work experiences in a group of non-UK EEA trained anaesthetists in a London hospital: a qualitative study [34].	Qualitative	United Kingdom	This study explored the acclimatisation experience of EU doctors with qualifications in anaesthesia arriving in the United Kingdom to take up clinical employment in the NHS	Acclimatisation conceived of as transfer of clinical expertise was problematic for doctors who felt they lacked the right kind of support. Doctors sought different opportunities to share wider perspectives on care deriving from their previous experience.
Recertifying as a doctor in Canada: international medical graduates and the journey from entry to adaptation [22].	Qualitative	Canada	This study aims to describe the recertification training experiences of IMGs in Canada in order to help medical training programmes understand how to facilitate the integration of IMGs into recipient medical communities	4 themes that typified IMG recertification training experiences: training entry barriers; and a 3-phase process of loss, disorientation and adaptation. International medical graduates must complete this 3-phase process in order to feel fully integrated into their professional environments
